# Extracellular loops matter – subcellular location and function of the lysine transporter Lyp1 from *Saccharomyces cerevisiae*

**DOI:** 10.1111/febs.15262

**Published:** 2020-03-11

**Authors:** Joury S. van‘t Klooster, Frans Bianchi, Ruben B. Doorn, Mirco Lorenzon, Jarnick H. Lusseveld, Christiaan M. Punter, Bert Poolman

**Affiliations:** ^1^ Department of Biochemistry Groningen Biomolecular Sciences and Biotechnology Institute University of Groningen The Netherlands

**Keywords:** amino acid, APC superfamily, loop, membrane transport, yeast

## Abstract

Yeast amino acid transporters of the APC superfamily are responsible for the proton motive force‐driven uptake of amino acids into the cell, which for most secondary transporters is a reversible process. The l‐lysine proton symporter Lyp1 of *Saccharomyces cerevisiae* is special in that the Michaelis constant from out‐to‐in transport ( $K_{m}^{\text{out}\to \text{in}}$ ) is much lower than $K_{m}^{\text{in}\to \text{out}}$ , which allows accumulation of l‐lysine to submolar concentration. It has been proposed that high intracellular lysine is part of the antioxidant mechanism of the cell. The molecular basis for the unique kinetic properties of Lyp1 is unknown. We compared the sequence of Lyp1 with APC para‐ and orthologues and find structural features that set Lyp1 apart, including differences in extracellular loop regions. We screened the extracellular loops by alanine mutagenesis and determined Lyp1 localization and activity and find positions that affect either the localization or activity of Lyp1. Half of the affected mutants are located in the extension of extracellular loop 3 or in a predicted α‐helix in extracellular loop 4. Our data indicate that extracellular loops not only connect the transmembrane helices but also serve functionally important roles.

## Graphical abstract

We performed alanine‐scanning mutagenesis of the extracellular loops of the yeast lysine transporter Lyp1. We find positions that affect either the localization or activity of Lyp1. Half of the affected mutants are located in the extension of extracellular loop 3 or in a predicted α‐helix in extracellular loop 4. We conclude that extracellular loops of amino acid transporters of the APC superfamily serve functionally important roles.

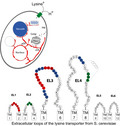

**Abbreviations**
APC: amino acid–polyamine–organocation
BATs: bacterial amino acid transporters
cER: cortical endoplasmic reticulum
EL: extracellular loop
MFS: major facilitator superfamily
NSS: neurotransmitter sodium symporter
pER: perinuclear endoplasmic reticulum
PM: plasma membrane
TMHs: transmembrane α‐helices
YATs: yeast amino acid transporters

## Introduction

The transport of amino acids in *Saccharomyces cerevisiae* is facilitated by yeast amino acid transporters (YATs) 1, which are members of the APC superfamily. The transport of amino acids is part of the cell's nitrogen regulation and biosynthesis pathways 2. We focus on the transport of basic amino acids which affects protein synthesis, oxidative stress tolerance, and possibly protein breakdown through effects on ubiquitination 3, 4. Basic amino acids are transported by only a subset of YATs, that is, the proteins encoded by Gap1, Hip1, Alp1, Can1, and Lyp1. Biochemical analyses have revealed the importance of cytoplasmic loop regions in these transporters in amino acid specificity 5, 6, endocytic recognition 7, 8, and trafficking 9, 10, 11, but systematic analysis of the extracellular loops has so far been unexplored in any YAT. At present, there is no structure of a YAT available, but models have been built on the basis of bacterial APC structures. Ghaddar *et al*. 12 were able to re‐engineer the specificity of Can1 from Arg to Lys on the basis of the structure of AdiC. Modeling of extracellular loop regions is more challenging as they are often not well resolved in crystal structures and are typically shorter in prokaryotic homologues and thus unique for eukaryotic membrane proteins 13, 14.

The lysine permease Lyp1 from *S. cerevisiae* is characterized by asymmetric transport kinetics, which is thought to form the basis for the massive accumulation of lysine 15. Although differences in kinetics of out‐to‐in *versus* in‐to‐out transport have been also observed for the lactose transporters LacY and LacS, they are much smaller than in Lyp1 and do not lead to an apparent unidirectionality of transport 16, 17. The asymmetric transport kinetics has recently been connected to the antioxidant strategy of the cell 3. High intracellular lysine triggers a reprogramming of redox metabolism, that is the glutathione concentrations increase, the levels of reactive oxygen species reduce and the oxidant tolerance of the cell increases 3. Given the important role of lysine, in particular import of lysine, in the physiology of the cell, we analyze Lyp1 and the features that set this protein apart from other (basic) amino acid transporters in yeast and beyond. Currently, only a few studies report specific functions for extracellular loops in APC proteins 18, 19, 20, 21, 22, 23, and they show roles for substrate recognition and gating by intramolecular anchoring. We now analyze the functional roles of extracellular loops in Lyp1 of *S. cerevisiae* by systematically substituting triplets of amino acids and determining the effects of the modifications on cellular location and translocation kinetics.

## Results

### Modeling and bioinformatic analysis of loop regions

Transmembrane α‐helices (TMHs) can be predicted with relative high accuracy, using topology prediction programs and multiple sequence alignments of homologous proteins, but loop regions, which often vary a lot in length and structure, are difficult to analyze without proper template. We used evfold, a program that exploits a maximum entropy analysis of the sequences of a protein family to determine evolutionary co‐variation in pairs of amino acid residues at specific sequence positions. Pairs of co‐evolved residues are then used as distance constraints to fold the protein of interest using the modeling software CNS (for a review see Ref. 24 for details). To validate the structural model, we benchmarked the loop regions obtained by evfold against a subset of topology and secondary structure predictors (Fig. S1).

**Fig. 1:**
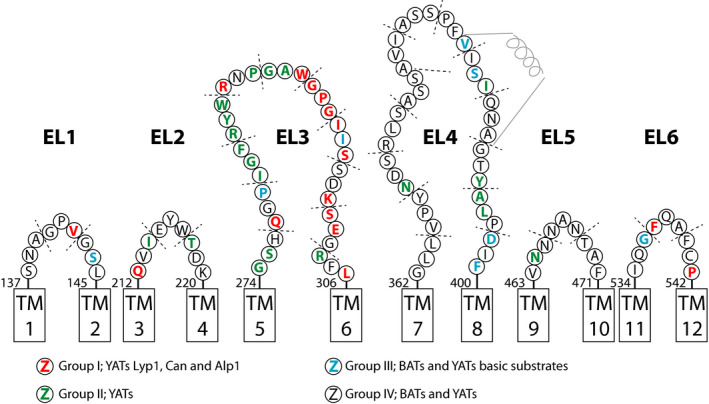
Topology model highlighting the extracellular loops of Lyp1 as predicted by evfold. Each circle represents an amino acid indicated by its one‐letter code. Color coding represents the conservation of each amino acid with respect to a category. Group I: YATs Lyp1, Can1, and Alp1, red. Group II: YATs, green. Group III: BATs with a substrate specificity toward basic amino acids, blue; and Group IV: loop regions that are less than 50% conserved in any of the homologs, white. TM, transmembrane segment. Start and end sequence numbering of each EL is indicated above the TM boxes.

Comparing Fig. 1 and Fig. S1, we find that the annotation of loop regions differs slightly depending on the prediction tool used. For instance, regions 140–142 GPV and 143–145 GSL of EL1 and 212–214 QVI of EL2 are annotated as part of TM1 and TM2, respectively, by the topology predictors. Similarly, evfold and the topology and secondary structure analysis suites predict the start of EL4 and most of EL6 differently. The evfold model is in line with the 3D model of Can1 12; a sequence alignment of both models is shown in Fig. S2. Noticeable is the similarity of both models in predicting the extracellular loops; minor differences are marked by color coding of the amino acid residues. Furthermore, we emphasize the prediction of an α‐helical structure in the middle of EL4.

**Fig. 2:**
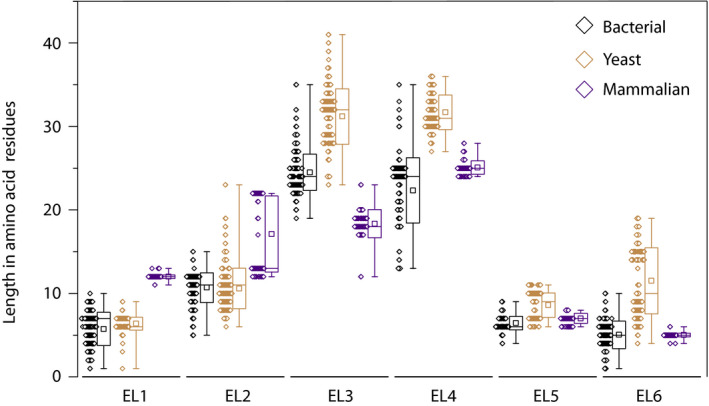
Bioinformatic analysis of extracellular loop (EL) length of bacterial, yeast, and mammalian amino acid transporters. The length of amino acid residues as function of each EL is given. The box plot displays the mean (square), median (horizontal line), standard deviation (box), and min/max values (whiskers) of each population. The distribution of the population of sequences is given left of the box plot. Here, each diamond represents a sequence that is binned with a size of 2. *n* = 835 for bacterial, *n* = 450 for yeast, and *n* = 105 for mammalian sequences.

We used the annotation predicted by evfold and the sequence conservation of bacterial and yeast amino acid transporters (BATs and YATs) from the APC superfamily to design mutations in the extracellular loops. For the analysis of amino acid conservation in extracellular loops, we focus on homologs with a sequence identity of 50% or more. We generated three consensus sequences from homologs of (a) YATs Lyp1, Can1, and Alp1; (b) homologs of BATs with basic amino acids as substrates; and (c) homologs of YATs with non‐basic amino acids as substrates. Next, we aligned those consensus sequences against the sequence of Lyp1 and annotated an amino acid residue as conserved in either of the following four groups (Fig. 1): Group I*:* YATs Lyp1, Can1, and Alp1 (red); Group II*:* YATs (green); Group III: BATs with basic amino acids as substrates (blue); and Group IV: all YATs and BATs with less than 50% conservation in any of the homologs (white). Noticeable is the string of colored residues in EL3 and EL4. The blue colors in EL4 indicate that these amino acids are conserved in both yeast and bacterial basic amino acid transporters, whereas the continuous string of red and green in EL3 suggests conservation in YATs solely (72% of the amino acids in EL3). Furthermore, when we align amino acid transporters from yeast (450 sequences) with mammalian (105 sequences) and bacterial (835 sequences), we find yeast transporters to be the largest (587 ± 26 amino acids) followed by mammalian (510 ± 15 amino acids) and bacterial (475 ± 20 amino acids) proteins. N termini and C termini of yeast and mammalian amino acid transporters are longer compared with those of bacteria and known to play a role in transporter regulation 25. However, EL3 and EL4 are significantly longer in yeast transporters, while EL1 and EL2 are longer in mammalian transporters (Fig. 2). EL6 displays two populations similar to EL2 in mammals: one having similar lengths and one with longer lengths. Here, the longer EL6 and EL2 of APC members in yeast and mammals are all proteins with high sequence identity to the general amino acid transporter/transceptor Gap1 and the large neutral amino acid transporter Lat1, respectively.

### Design and localization of Lyp1 mutants

**Fig. 3:**
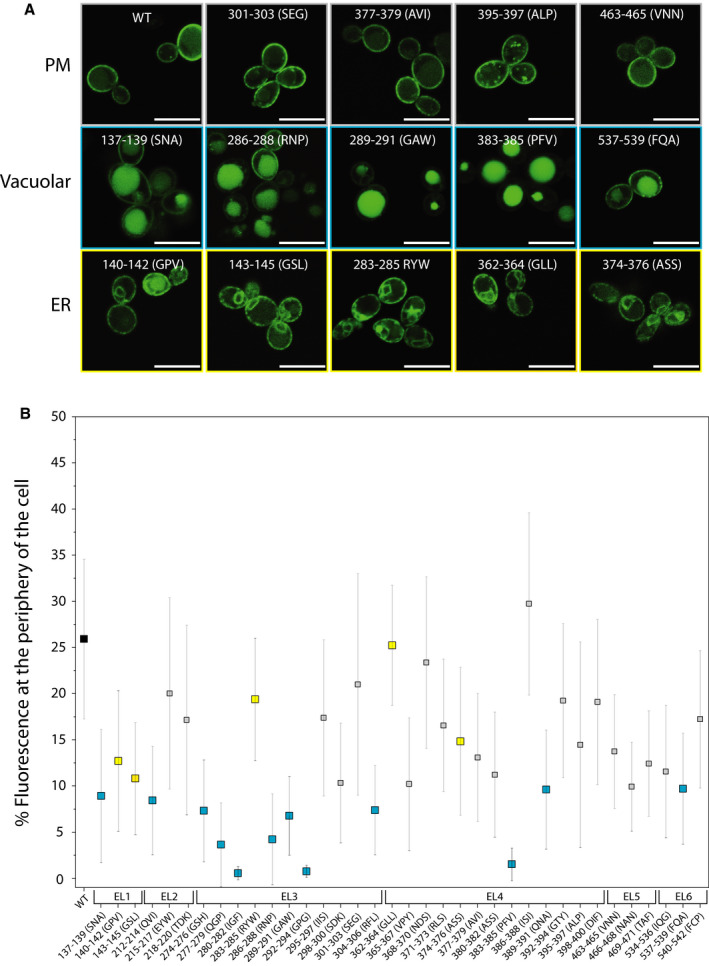
Percentage Lyp1‐YPet fluorescence at the cell periphery. Panel A: fluorescence microscopy images showing the subcellular location of a subset of Lyp1‐YPet mutants to the PM, vacuole, or ER. Panel B: % fluorescence at the periphery of *Saccharomyces cerevisiae*, expressing Lyp1, for the wild‐type protein (black square) and each mutant. Mutants displaying less than 10% peripheral fluorescence compared with wild‐type protein are colored blue. Mutants with apparent endoplasmic reticulum staining are colored yellow. Mutants with fluorescence staining between 10 and 35% and more similar to wild‐type Lyp1 are indicated in gray. Each value is determined from three biological replicates each with at least 50 cells. *n* = 3, error bars are standard error of the mean. Scale bars are 10 µm.

On the basis of the *in silico* analysis, we designed a set of mutants where in each case triplets of amino acids were changed into alanine; this approach is similar to the one described by Merhi *et al*. 26. In Fig. 1, each triplicate is indicated by a dashed line. To determine whether or not mutations affect the internal trafficking or folding of the transporters, we monitored the localization of Lyp1‐YPet in the cell (Fig. S3) and quantified the presence of Lyp1 in the PM by plotting the ratio of Lyp1‐YPet fluorescence at the periphery of the cell over the total fluorescence. We find twelve mutants with significantly increased internal fluorescence, most likely corresponding to vacuolar localization 27 (Fig. 3, blue squares). A caveat of conventional light microscopy is that the resolution is too low to discriminate cortical endoplasmic reticulum (cER) from the PM. Hence, fluorescence at the cell periphery does not unambiguously mean plasma membrane (PM) localization, although the presence in the cER yields discontinuous fluorescence unlike a localization in the PM 28, 29. By comparing our microscopy images with ER staining from literature 30, we assign five additional Lyp1 mutants that are localized in the cortical and perinuclear ER (Fig. 3, yellow squares).

### Transport activity of extracellular loop mutants

**Fig. 4:**
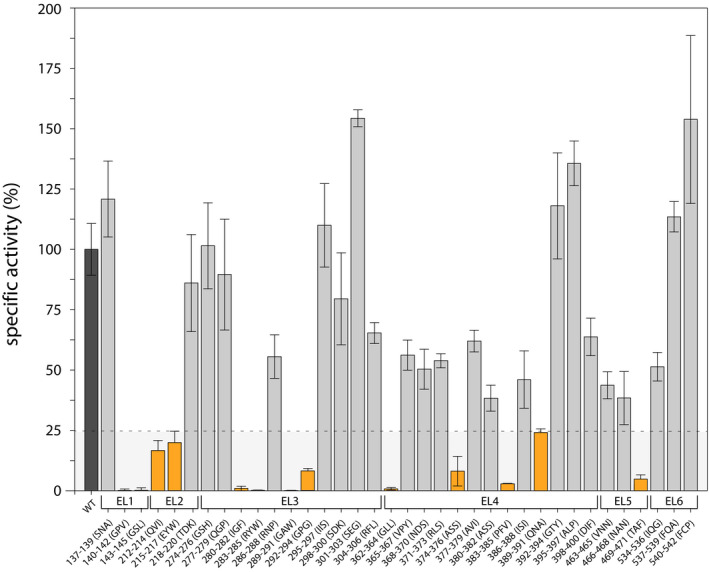
Transport activity of extracellular loop mutants of Lyp1. Dotted line separates mutants with less than 25% of wild‐type activity (light gray area and orange bars). *N* = 3, error bars are standard error of the mean.

In total, we find 17 mutants where the majority of Lyp1 molecules is mislocalized, most are found in EL3; five mutants have a localization similar to wild‐type Lyp1 (PM fluorescence level of 20–35%); and fourteen mutants have PM staining of 10–20%. Next, we performed transport assays at a substrate concentration of 20 µm (the *K*_m_ of wild‐type Lyp1 for l‐lysine ≈ 10 µm) and we normalized the transport rates for the number of cells and average absolute fluorescence at the cell periphery. This results in a specific activity that we express as a percentage relative to wild‐type Lyp1 (Fig. 4). We have set an arbitrary threshold at 25% of wild‐type Lyp1‐YPet activity. Of the 17 mislocalized mutants, we find 11 are below the threshold, of which six are completely inactive. The proteins with apparent peripheral plus ER staining fall in the class without activity, which is consistent with the notion that these proteins are not present in the PM. The activity of 11 mutants is comparable to that of the wild‐type, and twelve are significantly lower than the wild‐type protein but well above the threshold. Of the mutants with a wild‐type‐like location, 215–217 EYW is highly compromised in its transport (specific activity of 20%) and is situated in EL2. Of the proteins with intermediate fluorescence (level of 10–20%), 469–471 TAF is highly affected in transport (specific activity of 5%) and is present in EL5. Intriguingly, the mutants 277–279 QGP and 286–288 RNP with less than 5% peripheral fluorescence show wild‐type transport (specific activity of 90% and 55%, respectively), that is after normalization for the amount of protein in the membrane. This suggests that these mutants reach the PM, but are quickly sorted to the vacuole. Overall, our results show that 15 out of 36 mutants display reduced transport activity and/or mislocalization of the protein (Table S3), emphasizing the importance of the extracellular loop regions.

### Transport kinetics of extracellular loop mutants

**Fig. 5:**
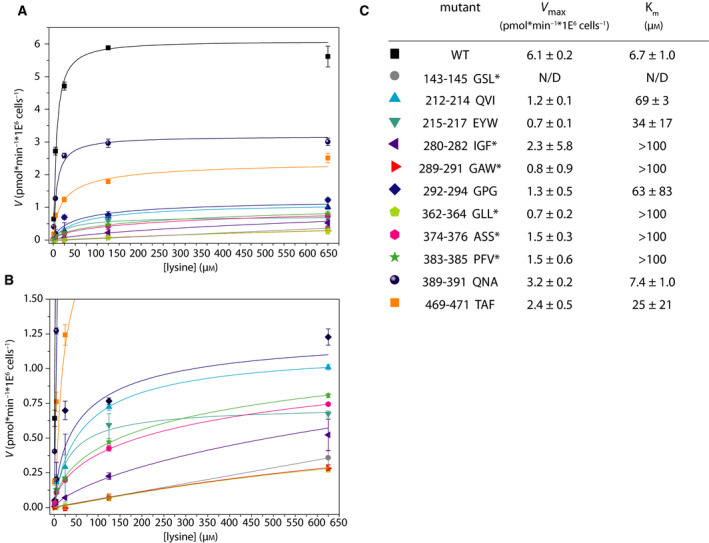
*V*_max_ and *K*_m_ of Lyp1 mutants. Panel A: initial rate of transport (*V*) as a function of lysine concentration for a selected set of mutants. Panel B: zoom‐in of Panel A. Panel: estimated *V*_max_ and *K*_m_ values by fitting the data to the hyperbolic function: *V* = (*V*_max_ × [*S*])/(*K*_m_ + [*S*]). Here, *V* = velocity, [*S*] = substrate concentration, *V*_max_ = maximal velocity, and *K*_m_ = [*S*] at which *V* = 1/2*V*_max_. ND, not determined. *underestimated values because we cannot discriminate the fraction of Lyp1 in the plasma membrane from the fraction in the ER. *n* = 3, error bars are standard error of the mean.

We hypothesized that given mutants may be affected in the translocation kinetics for lysine transport and have an altered *V*_max_ and/or affinity constant for transport (*K*_m_). We obtained estimates of *V*_max_ and *K*_m_ values for 11 out of 13 mutants (Fig. 5B,C); for 140–142 GPV and 283–285 RYW, the activity was essentially zero, consistent with their localization in the ER. We find a reduced *V*_max_ for the remaining 11 mutants, but for six mutants the rates may be underestimated because part of the protein is retained in the ER (asterisks Fig. 5C). Hence, we were unable to make the appropriate correction for the fraction of the protein in the plasma membrane. Together, these results indicate that the *K*_m_ is increased by 5 to more than 10‐fold for the majority of mutants. In case of 143–145 GSL, the rate of transport increased linearly up to a concentration of 650 µm, indicating that the *K*_m_ may even be higher.

**Fig. 6:**
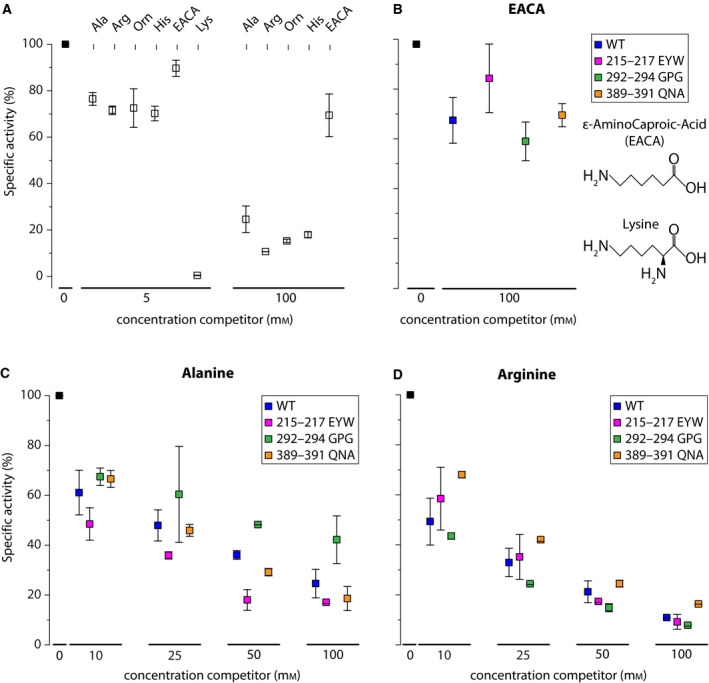
Transport activity of Lyp1 in the absence and presence of competing substrate. Panel (A) specific activity of WT Lyp1 as a function of competitor concentration. Ala, alanine; Arg, arginine; Orn, ornithine; His, histidine; EACA, ε‐aminocaproic acid; Lys, lysine. Panel (B) specific activity of WT and Lyp1 mutants in the absence and presence of EACA. Panel (C) specific activity of WT and Lyp1 mutants as a function of the alanine concentration. Panel (D) specific activity of WT and Lyp1 mutants as a function of alanine concentration. The square represents the average of two biological replicates (*n* = 2). Error bars indicate the maximum and minimum value.

Next, we performed substrate competition experiments to determine whether mutants with altered affinity constants for lysine uptake are affected in their substrate specificity. We focused on mutants with sufficient residual transport activity to enable accurate measurements of substrate competition, using [^14^C]‐lysine as reporter substrate. We tested the amino acids alanine (neutral), arginine, ornithine, and histidine (basic), and deaminated lysine and ε‐aminocaproic acid (EACA) as competitors. For wild‐type Lyp1, we find that all amino acids show minor competition with [^14^C]‐lysine at 250‐fold excess (20 µm [^14^C]‐lysine plus 5 mm of competitor), but 100 mm of unlabeled amino acids reduced the specific activity of lysine transport by > 80% (Fig. 6A). As anticipated, basic amino acids compete more strongly than alanine and ε‐aminocaproic acid is not really a substrate of Lyp1 (Fig. 6B); a 250‐fold excess of unlabeled lysine was included as control, which shows the expected apparent inhibition. Based on these results, we chose alanine (Fig. 6C) and arginine (Fig. 6D) for further studies with the mutants 215–217 EYW, 292–294 GPG, and 389–391 QNA. We find for all mutants that both alanine and arginine are inhibiting the uptake of lysine and that the degree of inhibition is similar to that of wild‐type Lyp1. We thus conclude that the mutants are not affected in their substrate specificity.

## Discussion

**Fig. 7:**
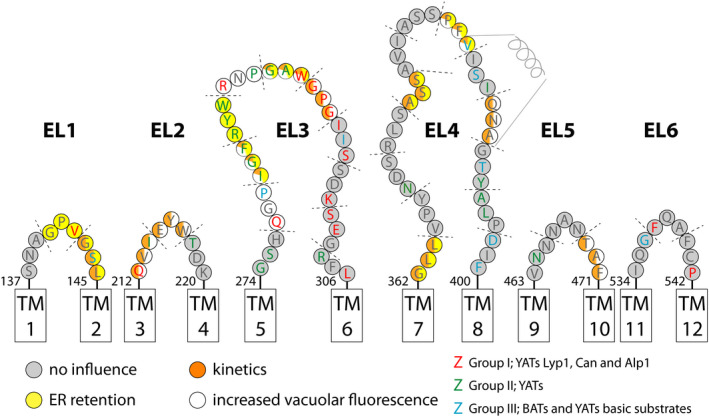
Summary of the data projected on the topology model of extracellular loops of Lyp1. Each circle represents an amino acid indicated by its one‐letter code. Letter color coding represents the conservation of each amino acid with respect to a group as presented in Fig. 1. Triplicate mutants are separated by dotted lines, and their circled amino acids are colored according to their phenotype. No influence (gray), retention in the ER (yellow), altered kinetic parameters (orange), and increased vacuolar fluorescence (white). Mixed colors indicate a plural effect.

Figure 7 and Table S3 summarize the experimental findings. We categorize the mutations into four groups: (a) no influence (gray); (b) changed kinetics (orange); (c) ER retention (yellow); and (d) increased vacuolar localization and breakdown of the protein (white). We find that mutants affected in transport also display increased vacuolar fluorescence (orange/white), which suggests that they make it to the plasma membrane but are more rapidly turned over. Most of these proteins are obtained with mutations in EL2, EL4, and EL5. Yellow/orange circled mutants show apparent ER localization, but they are partially active (specific activity of 5–25% relative to wild‐type), indicating that a fraction of these proteins is localized to the PM. The ER retention suggests that these proteins are affected in trafficking to the plasma membrane. This phenotype is mostly found for mutants in EL1, EL3, and EL4. Two mutants (277–279 QGP and 286–288 RNP) located in EL3 show increased vacuolar fluorescence and low peripheral staining, but the kinetic parameters of transport are not much altered (white circles). Another two mutants (280–282 IGF and 289–291 GAW) situated in EL3 show altered kinetics in combination with increased vacuolar and ER staining (white/yellow/orange), suggesting that these proteins are affected in their trafficking and possibly their stability but once inserted in the plasma membrane they are active. Two more mutants (140–142 GPV and 283–285 RYW) in EL1 and EL3 show no activity, which is consistent with their ER (yellow circles). Finally, mutations in EL6 have no effect on transport, even though 1/3 of the residues are conserved in the basic amino acid transporters of YATs or BATs.

Thus, we find regions in extracellular loops of the yeast lysine proton symporter Lyp1 that play a role in localization and or specific activity of the protein; the mutants have a reduced affinity for lysine, but they are not affected in their substrate specificity. We do not find a correlation between conservation of residues and the severity of the mutations on transport or localization. However, 50% of the mutations that display an effect are located in EL3, which is extended and highly conserved in yeast APC transporters but not in bacterial or in mammalian homologs. Nine out of fifteen affected mutants contain a glycine, proline, or both. These amino acids are often part of turns or otherwise critical in the structures of proteins, and substituting those for alanine might disrupt turn formation or structures associated with stability or activity. Of the remaining six mutants, two in EL2 and one in EL5 are at the boundary of TMHs: One is located in the predicted α‐helix and the other two in the middle of EL3 and EL4.

What do we know of extracellular loop regions in membrane transporters in general and in members of the APC superfamily specifically? Extracellular loop regions are shorter than cytoplasmic loops, and very few functions other than N‐linked or O‐linked glycosylation are associated with these protein parts 31. However, some functional roles for extracellular loops in proteins not part of the APC superfamily have been described. These functions range from substrate recognition and binding, as well as protein dimerization and internal trafficking as shown for the MFS‐superfamily transporters Oct1 from rats 32 and humans 33 and Hup1/2 from *Chorella kessleri* 34, gating functions and conformational changes involved in the transport process for glutamine transporters ASCT2 from human 35 and Glt_PH_ from *Pyrococcus horikoshii* 36, and efficient export of substrates for the ABC‐multidrug exporter Pdr5 from *S. cerevisiae* 37.

For APC‐superfamily members, the structure of the NSS family (subfamily of the APC superfamily) bacterial sodium‐coupled amino acid transporter ‘LeuT’ serves as the paradigm. Similar to YATs and BATs, extracellular loops of eukaryotic NSS‐family homologs are typically longer than those of LeuT 13, 14. Functional evidence for the importance of extracellular loops in eukaryotic APC‐superfamily members comes from elaborate analysis of the NSS‐family human serotonin transporter SERT 38, 39; EL2 interacts with other extracellular loops or TMHs and is important for transport 18; EL4 and EL5 are important for protein assembly and stability; and EL5 has a role in ion‐flux coupling and forms part of the external gate 40. Similar roles in gating have also been reported for other APC‐superfamily members 20, 21, 22. Furthermore, substrate discrimination by EL5 has been described for the γ‐aminobutyric acid (GABA) transporter GAT1 from mice 19 and the length of EL5 affects translocation rates 23. Noticeable is that none of these studies in mammalian and bacterial transporters report roles for EL3, the regions where we find the strongest effects on transport and trafficking.

For the yeast APC‐family members, Gap1 41, Tat2 42 Bap2 43, Can1 12, and PrnB 44, 45 homology models have been constructed on the basis of the crystal structure of the arginine/agmatine antiporter ‘AdiC’ from *Escherichia coli* 46 *.* Although modeling of loops and termini was incomplete, the models identified the substrate‐binding site, the origin of substrate specificity, and the role of some intracellular loops herein 5, 26. Two studies report mutations in EL4 of Gap1 6 and Can1 5 that changed substrate specificity. Strikingly, mutations in Gap1 are in the region of EL4 that is predicted to form an α‐helical structure similar to what we and others find for Lyp1, SERT 38, and LeuT 47.

What can we conclude on the basis of our results for Lyp1? We find 50% of the mutations in EL3 to impair localization and or specific activity of the protein. Full or partial impaired exocytic trafficking (ER fluorescence), increased endocytic turnover (vacuolar fluorescence), and altered *V*_max_ and/or *K*_m_ are found for mutations in this region. Thus, extracellular loops in yeast APCs are not merely TMH connecting structures but serve important functional and structural roles, as here shown for Lyp1.

## Materials and methods

### Plasmid and strain construction

The strains and plasmids used in this study are listed in Tables S1 and S2, respectively. All plasmids were generated using uracil excision‐based cloning 48. The amplification of DNA with uracil‐containing primers was performed using the polymerase PfuX7 49. Amplified fragments were assembled into full plasmids by treatment with DNA glycosidase and DNA glycosylase‐lyase endo VIII, commercially available as ‘USER’, following the manufacturer's instructions (New England Biolabs, Ipswich, MA, USA). The constructs were transformed into *E. coli* MC1061 by the heat‐shock procedure. Subsequently, plasmids were isolated using a plasmid extraction kit (Macherey‐Nagel, Düren, Germany) and the DNA was verified by sequencing. Plasmids were subsequently transformed into *S. cerevisiae* using the LiAc method 50. Downstream selection of monoclonal *S. cerevisiae* was based on protein expression by selecting clones that show a homogenous distribution and high intensity of YPet, using a flow cytometer (BD Accuri™, Durham, NC, USA) equipped with a 488‐nm laser.

### Preparation of* S. cerevisiae* cells for *in vivo* transport assays and fluorescence imaging

All chemicals were purchased from Sigma‐Aldrich (Darmstadt, Germany), unless otherwise indicated. Cells were cultured at 30 °C with 200 RPM shaking in 50 mL CELLreactor™ filter top tubes (Greiner Bio‐On, Kremsmünster, Austria). Strains were grown overnight by inoculation in 5‐mL synthetic glucose media without uracil and lysine and supplemented with the dipeptide Lys‐Lys. Media were prepared by dissolving 2% w/v glucose and 0.69% w/v yeast nitrogen base (YNB) without amino acids (Formedium, Norfolk, UK). Media were supplemented with 0.19% w/v Kaiser synthetic mixture without uracil and lysine 51 *,* that is, a mixture containing 18 mg·L^−1^ adenine, 76 mg·L^−1^ myo‐inositol, 8 mg·L^−1^ para‐aminobenzoic acid, and 76 mg·L^−1^ of all 20 standard amino acids (l‐leucine was added at 380 mg·L^−1^) except l‐lysine (Formedium). Finally, 200 mg·L^−1^ Lys‐Lys was added. Subcultures were grown and diluted for two to three consecutive days such that the OD_600_ never exceeded 1. Cells were centrifuged at 3000 ***g*** for 5 min at 4 °C, supernatant was decanted, and cells were suspended in ice‐cold 100 mm potassium phosphate, 10 mm glucose, pH 6.0. This step was performed twice before suspension of the cells to an OD_600_ of 5.

### Quantitative fluorescence imaging

Quantitative fluorescence live‐cell imaging was performed on a LSM 710 commercial scanning confocal microscope (Carl Zeiss MicroImaging, Jena, Germany), equipped with a C‐Apochromat 40×/1.2 NA objective, and blue argon ion laser (488 nm). Frame size was 1024 × 1024; bit depth, 16 bit; and pixel size, 10 µm. Pinhole was set to 1.0 (arbitrary unit). Laser power, gain, and zoom were kept constant for all images. Cells were immobilized between a glass slide and coverslip. Images were acquired with the focal plane positioned at the midsection of the cells. Acquired images were processed using imagej/fiji 52. The outline of the cell was selected to determine the fluorescence in the plasma membrane (PM), from which the area of the cell and mean intensity per pixel of the selection were generated. We excluded values ±2 times the standard error of the mean (SEM). The percentage of Lyp1 in the PM was calculated by taking the ratio of fluorescence in the PM over that of the whole cell. The mean intensity/pixel of the selection of PM fluorescence was used for normalizing transport data (next section).

### *In vivo* transport assays

Each assay contained cells at OD_600_ of 0.5. All transport assays were performed in 5‐mL glass tubes placed in a water bath, and the solution of each tube was stirred magnetically. Each glass tube contained 100 mm potassium phosphate, 10 mm glucose, pH 6.0 at 30 °C, using 20 µm l‐[^14^C(U)]‐lysine (unless otherwise indicated) and a magnetic stirrer bar. Samples were mixed by magnetic stirring in a total volume of 525 µL. At given time intervals, 100 µL samples were taken and quenched in another glass tube containing 2 mL ice‐cold ‘stop’ buffer of the same composition as described above, but without lysine. Cells were rapidly separated from external buffer by ultrafiltration, using a setup where a filter holder was placed on top of a container that was connected to a vacuum pump. Cells were collected onto a 0.45‐µm pore size nitrocellulose filter (GE Healthcare, Little Chalfont, UK) and washed with another 2 mL of stop buffer. Filters were transferred in Eppendorf tubes and dissolved using 2 mL of scintillation solution (Emulsifier^plus^; PerkinElmer, Waltham, MA, USA) and vortexed before radioactivity was determined by liquid scintillation counting (Tri‐Carb 2800TR Liquid Scintillation Analyzer; PerkinElmer). The number of cells in each sample was counted using a flow cytometer (BD Accuri™), with the following settings: volume = 20 µL, flow rate = medium, and OD_600_ of 0.125. The acquired transport data were normalized for protein quantity using the mean intensity/pixel of the selection (PM fluorescence) and for cell number determined from the flow cytometry data. Transport rates were estimated from the slope of the linear part of the progress curves, using the integrated ‘linest’ function in Excel. All other analyses (e.g., curve fitting, statistics) were performed with standard functions in Origin (OriginLab, Northampton, MA, USA). Each experiment was performed in triplicate using biological replicates. For *K*_m_ and *V*_max_ measurements, [^14^C]‐lysine concentrations of 1, 5, 25, 125, and 625 µm were used.

### Topology modeling

A structural model of Lyp1 was generated using the evfold prediction software 24, 53, 54. The following parameters, deviating from the default settings, are as follows: protein: UniProt accession no. P32487; α‐helical TMM domain: yes; Pfam member selector: PF00324; minimum sequence identity: 20%; membrane topology override: topcons (http://topcons.cbr.su.se) 55; membrane topology prediction: default settings; and input: UniProt accession no. P32487. The retrieved PDB file was analyzed using pymol (The PyMOL Molecular Graphics System, Version 1.5.0.4 Schrödinger, LLC, Mannheim, Germany).

Benchmarking of the evfold prediction by secondary structure predictors was done using phyre2 56 *,* raptorx 57, and i‐tasser 58 and by topology predictors: hmmtop 59, tmpred 60 *, *Predicts protein 61, and topcons 55 *;* the output of the latter program is a consensus based on polyphobius, octopus, phillius, scampi, and spoctopus. A one was assigned if an amino acid was predicted to be part of an α‐helix or transmembrane segment. If not, a zero was assigned. The sum of ones and zeros was plotted as a function of each amino acid and used to evaluate the quality of the evfold model.

### Amino acid conservation in YATs and BATs

Unique sequences were obtained from the UniProt 62 PF00324 family and grouped on the basis of their sequence similarity to either Lyp1 from *S. cerevisiae*, LysP from *Salmonella typhimurium,* or *S. cerevisiae* core AAPs according to Ljungdahl and Daignan‐Fornier 63 (excluding Lyp1, Can1, and Alp1). For Lyp1 and LysP, homologs were included when annotated in UniProt as basic amino acid transporters and having 52–99% sequence identity with Lyp1 or LysP, which yielded 68 and 482 sequences, respectively. Homologs of the *S. cerevisiae* members of the AAP core cluster were acquired using UniRef50 62 groups, P19145, P48813, P38084, P06775, Q08986, P38085, P38967, P15380, P43548, P53388, P38090, and Q03770, corresponding to reference sequences of Gap1, Gnp1, Bap2, Hip1, Sam3, Tat1, Tat2, Put4, Agp3, Dip5, Agp2, and Ssy1, respectively. Each UniRef50 group was trimmed to 90% identity of the reference sequences, resulting in 460 sequences. Next, for each of the three groups a consensus sequence was obtained in jalview 64, which was based on a multiple sequence alignment generated by clustal omega 65. Then, the corresponding consensus sequences were aligned against the sequence of Lyp1 and gaps with respect to Lyp1 were removed. The aligned sequences were plotted in Microsoft Excel, and conservation of each amino acid with respect to one another was determined. We considered a valid conservation if the most frequent residue in one consensus sequence matched the first or second most conserved residue in the other. Based on this, each amino acid in the sequence of Lyp1 was annotated as conserved in one of four groups: (a) yeast basic amino acid transporters Lyp1, Can1, and Alp1; (b) yeast amino acid transporter excluding Lyp1, Can1, and Alp1; (c) bacterial basic amino acid transporters and yeast basic amino acid transporters Lyp1, Can1, and Alp1; and (d) yeast and bacterial amino acid transporters (BAT).

### Bioinformatics analysis extracellular loop length of yeast and bacterial APC proteins

Homologous sequences of proteins belonging to the amino acid–polyamine–organocation ACP family (TCDB 2.A.3) of yeast and bacteria were obtained from UniProt 62. UniRef90 clusters were taken based on protein accession numbers: for yeast, P32487, P19145, P48813, P38084, P06775, Q08986, P38085, P38967, P15380, P53388, and P38090 corresponding to Lyp1, Gap1, Gnp1, Bap2, hip1, Sam3, Tat1, Tat2, Put4, Dip5, and Agp2, respectively; for mammalian, Q01650, Q9UHI5, and Q9UPY5 corresponding to Lat1, Lat2, and Xct, respectively; and for bacterial, P24207, P37460, P25737, P15993, P0AAE0, P25527, P77610, P39137, and P42087 corresponding to PheP, ProY, LysP, AroP, CycA, GabP, AnsP, RocE, and HutM, respectively. This resulted in a population of 450, 105, and 835 sequences for yeast, mammalian, and bacterial proteins, respectively. The topology of each sequence within the population was predicted using topcons 55. Next, using the topology information for each sequence, the number of amino acids comprising each extracellular loop was extracted. Finally, we plotted the values: average length, median, standard deviation, and minimal and maximal values of each extracellular loop from the yeast and bacterial APC proteins.

## Conflict of interest

The authors declare no conflict of interest.

## Author contributions

JSK, FB, and BP designed the research plan; JSK, FB, RBD, ML, JHL, and BP analyzed the data. CMP wrote scripts for data analysis, and JSK, RBD, ML, and JHL performed experiments. JSK and BP wrote the paper.

## Supporting information


**Fig. S1.** Topology and secondary structure prediction of Lyp1.
**Fig. S2.** Multiple sequence alignment of Can1 and Lyp1.
**Fig. S3.** Fluorescence microscopy images of Lyp1 wild‐type and mutants.
**Table S1.** Strains used in this study.
**Table S2.** Plasmids used in this study.
**Table S3.** Summary of experimental observations and conclusions.
