# Supplementary material for: Extracellular loops matter – subcellular location and function of the lysine transporter Lyp1 from Saccharomyces cerevisiae
Source: FEBS J. 2020 Mar 11;287(20):4401–14. doi: 10.1111/febs.15262 (PMC7687128; doi:10.1111/febs.15262)
Supplement: Supplementary file 1 — Fig. S1. Topology and secondary structure prediction of Lyp1. Fig. S2. Multiple sequence alignment of Can1 and Lyp1. Fig. S3. Fluorescence microscopy images of Lyp1 wild‐type and mutants. Table S1. Strains used in this study. Table S2. Plasmids used in this study. Table S3. Summary of experimental observations and conclusions. [file FEBS-287-4401-s001.zip › febs15262-sup-0001-Supinfo.pdf]

FEBS PRESS
science publishing by scientists
WILEY
The FEBS Journal
www.febsjournal.org

Extracellular loops matter – subcellular location and function of the lysine transporter Lyp1 from *Saccharomyces cerevisiae*

Joury S. van‘t Klooster, Frans Bianchi, Ruben B. Doorn, Mirco Lorenzon, Jarnick H. Lusseveld, Christiaan M. Punter and Bert Poolman

DOI: 10.1111/febs.15262

SUPPLEMENTARY INFORMATION

Extracellular loops matter: subcellular location and activity of the lysine transporter Lyp1 from *Saccharomyces cerevisiae*

Joury S van 't Klooster, Frans Bianchi, Ruben Doorn, Mirco Lorenzon, Jarnick Lusseveld, Christiaan M. Punter and Bert Poolman[1]

Department of Biochemistry, University of Groningen, Groningen Biomolecular Sciences and Biotechnology Institute, Nijenborgh 4, 9747 AG Groningen, The Netherlands

[1]To whom correspondence should be addressed E-mail: b.poolman@rug.nl

Supplementary Tables S1-S3
Supplementary Figures S1-S3

Table S1: Strains used in this study

strains	*genotype*	*reference*
E. coli MC1061	F - Δ(*araA-leu*)7697 [*araD*139]$_{B/r}$ Δ(*codB-lacI*)3 *gal*K16 *galE*15(GalS) λ - e14- *mcrA*0 *relA*1 *rpsL*150 *spoT*1 *mcrB*1 *hsdR*2	Casadaban 1980 et al[66]
S. cerevisiae *22Δ6AAL*	Sigma22574d *MATα gap1*Δ1 *put4*Δ1 *uga4*Δ1 Δ*can*1::*HisG* Δ*lyp1*::*HisG* Δ*alp1*::*His*G *lys2*::*HisG*	Fischer 2002 et al[67]

Table S2: Plasmids used in this study

Name	*Description*	*Reference*
pJK1000	prs316 lyp1-TEV-YPet-His$_8$ (lyp1 allel)	This Study
pJK1011	Ala substitution at position 137-139 (SNA) in pJK1000	This study
pJK1012	Ala substitution at position 140-142 (GPV) in pJK1000	This study
pJK1013	Ala substitution at position 143-145 (GSI) in pJK1000	This study
pJK1014	Ala substitution at position 212-214 (QVI) in pJK1000	This study
pJK1015	Ala substitution at position 215-217 (EYW) in pJK1000	This study
pJK1016	Ala substitution at position 218-220 (TDK) in pJK1000	This study
pJK1019	Ala substitution at position 274-276 (GSH) in pJK1000	This study
pJK1020	Ala substitution at position 277-279 (QGP) in pJK1000	This study
pJK1021	Ala substitution at position 280-282 (IGF) in pJK1000	This study
pJK1022	Ala substitution at position 283-285 (RYW) in pJK1000	This study
pJK1023	Ala substitution at position 286-288 (RNP) in pJK1000	This study
pJK1024	Ala substitution at position 289-291 (GAW) in pJK1000	This study
pJK1025	Ala substitution at position 292-294 (GPG) in pJK1000	This study
pJK1026	Ala substitution at position 295-297 (IIS) in pJK1000	This study
pJK1027	Ala substitution at position 298-300 (SDK) in pJK1000	This study
pJK1028	Ala substitution at position 301-303 (SEG) in pJK1000	This study
pJK1029	Ala substitution at position 304-306 (RFL) in pJK1000	This study
pJK1030	Ala substitution at position 362-364 (GLL) in pJK1000	This study
pJK1031	Ala substitution at position 365-367 (VPY) in pJK1000	This study
pJK1032	Ala substitution at position 368-370 (NDS) in pJK1000	This study
pJK1033	Ala substitution at position 371-373 (RLS) in pJK1000	This study
pJK1034	Ala substitution at position 374-376 (ASS) in pJK1000	This study
pJK1035	Ala substitution at position 377-379 (AVI) in pJK1000	This study
pJK1036	Ala substitution at position 380-382 (ASS) in pJK1000	This study
pJK1037	Ala substitution at position 383-385 (PFV) in pJK1000	This study
pJK1038	Ala substitution at position 386-388 (ISI) in pJK1000	This study
pJK1039	Ala substitution at position 389-391 (QNA) in pJK1000	This study
pJK1040	Ala substitution at position 392-394 (GTY) in pJK1000	This study
pJK1041	Ala substitution at position 395-397 (ALP) in pJK1000	This study
pJK1042	Ala substitution at position 398-400 (DIF) in pJK1000	This study
pJK1043	Ala substitution at position 463-465 (VNN) in pJK1000	This study

pJK1044	Ala substitution at position 466-468 (NAN) in pJK1000	This study
pJK1045	Ala substitution at position 469-471 (TAF) in pJK1000	This study
pJK1046	Ala substitution at position 534-536 (IQG) in pJK1000	This study
pJK1047	Ala substitution at position 537-539 (FQA) in pJK1000	This study
pJK1048	Ala substitution at position 540-542 (FCP) in pJK1000	This study

Table S3: Summary of experimental observations and conclusions.
*No significant influence on transport rate. Color-coding corresponds to that of figure 3 and 4 of the main manuscript.

EL	Mutant	V % compared to WT	Phenotype	Category
4	377-379 AVI	62	PM	*
5	463-465 VNN	44	PM	*
5	466-468 NAN	38	PM	*
3	304-306 RFL	65	PM/vesicular bodies	*
4	368-370 NDS	50	PM/vesicular bodies	*
4	395-397 ALP	136	PM/vesicular bodies	*
4	398-400 DIF	64	PM/vesicular bodies	*
1	137-139 SNA	121	PM/Vacuole	*
2	218-220 TDK	86	PM/Vacuole	*
3	274-276 GSH	102	PM/Vacuole	*
3	277-279 QGP	90	PM/Vacuole	*
3	286-288 RNP	56	PM/Vacuole	*
3	295-297 IIS	110	PM/Vacuole	*
3	298-300 SDK	80	PM/Vacuole	*
3	301-303 SEG	154	PM/Vacuole	*
4	365-367 VPY	56	PM/Vacuole	*
4	371-373 RLS	54	PM/Vacuole	*
4	380-382 ASS	38	PM/Vacuole	*
4	386-388 ISI	46	PM/Vacuole	*
4	392-394 GTY	118	PM/Vacuole	*
6	534-536 IQG	51	PM/Vacuole	*
6	537-539 FQA	114	PM/Vacuole	*
6	540-542 FCP	154	PM/Vacuole	*
**Mutants with changed kinetics compared to WT Lyp1**				
2	215-217 EYW	20	PM/Vacuole	V_{max} <10% & K_m 5-fold up
5	469-471 TAF	4	PM/cER/Vacuole	V_{max} <50% & K_m 5-fold up
1	140-142 GPV	0	pER/cER	V = 0 & ER retention
1	143-145 GSL	0	PM/pER/cER	K_m >100-fold up
3	283-285 RYW	0	pER/cER	V = 0 & ER retention
4	362-364 GLL	1	PM/pER/cER	K_m 100-fold up
4	374-376 ASS	8	PM/pER/cER	K_m 100-fold up
2	212-214 QVI	17	PM/Vacuole	V_{max} <50% & K_m 10-fold up
3	292-294 GPG	8	PM/cER/Vacuole	V_{max} <50% & K_m 10-fold up
3	280-282 IGF	1	PM/pER/cER/Vacuole	V_{max} <50% & K_m >100-fold up
3	289-291 GAW	0	PM/pER/cER/Vacuole	K_m >100-fold up
4	383-385 PFV	3	PM/pER/cER/Vacuole	V_{max} <50% & K_m >100-fold up
4	389-391 QNA	24	PM/Vacuole	V_{max} <50%

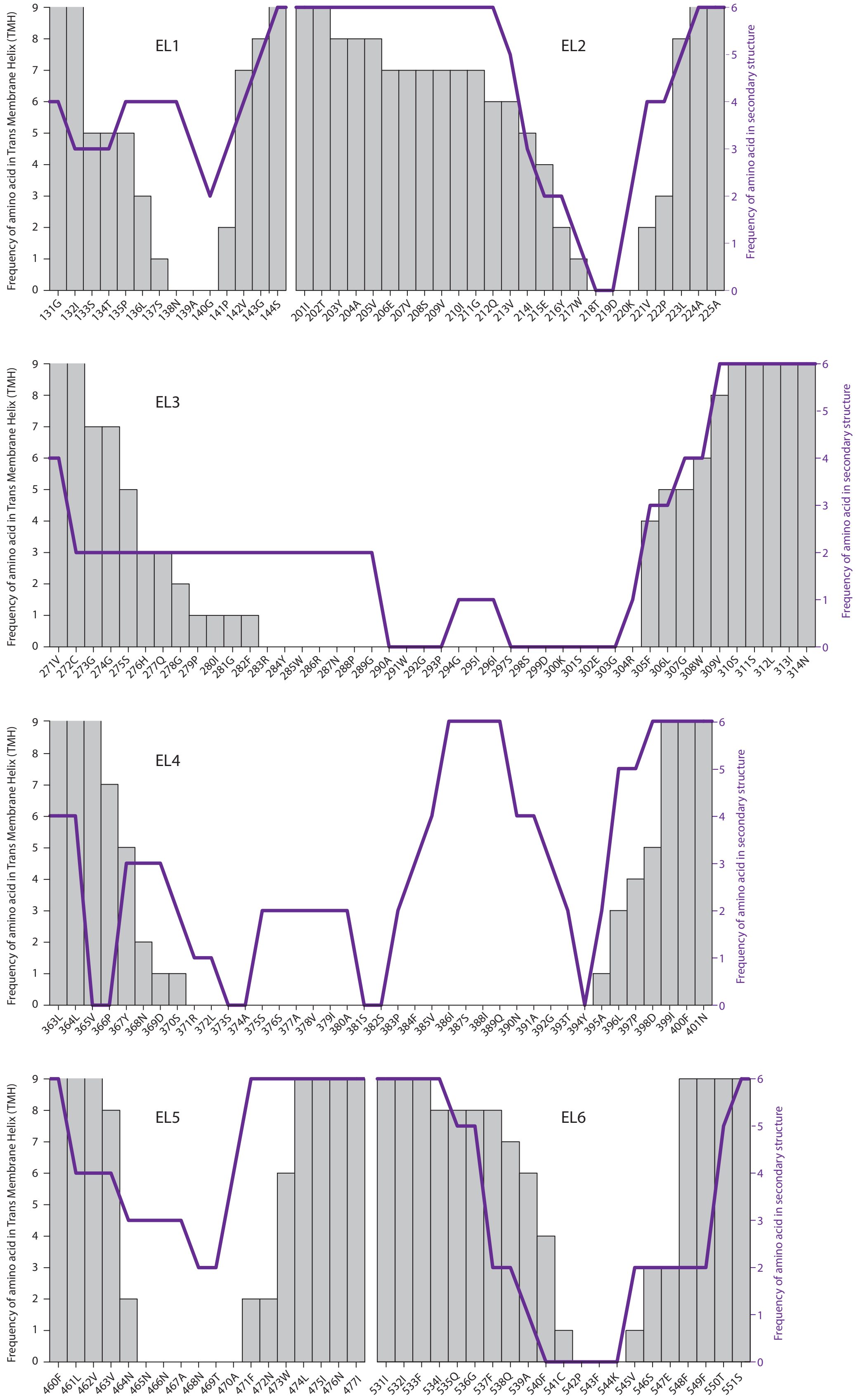

Figure S1: Topology and secondary structure prediction of Lyp1. Frequency of amino acids predicted to be in either a transmembrane segment (grey bars) or α–helix (purple line) as a function of amino acid residue number.

```
CAN1                 1 -------MTNSKEDA---DIEEKHMYNEPVTTLFHDVEASQTHHRRG--S     38
LYP1                 1 MGRFSNIITSNKWDEKQNNIGEQSMQELPEDQIEHEMEAIDPSNKTTPYS     50

CAN1                39 IPLKDEKSKE---------LYPLRSFPTRVNGEDTFSMEDGIG-DEDEGE     78
LYP1                51 IDEKQYNTKKKHGSLQGGAIADVNSITNSLTRLQVVSHETDINEDEEEAH    100

CAN1                79 VQNAEVKRELKQRHIGMIALGGTIGTGLFIGLSTPLTNAGPVGALISYLF    128
LYP1               101 YEDKHVKRALKQRHIGMIALGGTIGTGLFVGISTPLSNAGPVGSLIAYIF    150

CAN1               129 MGSLAYSVTQSLGEMATFIPVTSSFTVFSQRFLSPAFGAANGYMYWFSWA    178
LYP1               151 MGTIVYFVTQSLGEMATFIPVTSSITVFSKRFLSPAFGVSNGYMYWFNWA    200

CAN1               179 ITFALELSVVGQVIQFWTYKVPLAAWISIFWVIITIMNLFPVKYYGEFEF    228
LYP1               201 ITYAVEVSVIGQVIEYWTDKVPLAAWIAIFWVIITLMNFFPVKVYGEFEF    250

CAN1               229 WVASIKVLAIIGFLIYCFCMVCGAGVTGPVGFRYWRNPGAWGPGIISKDK    278
LYP1               251 WVASVKVLAIMGYLIYALIIVCGGSHQGPIGFRYWRNPGAWGPGIISSDK    300

CAN1               279 NEGRFLGWVSSLINAAFTFQGTELVGITAGEAANPRKSVPRAIKKVVFRI    328
LYP1               301 SEGRFLGWVSSLINAAFTYQGTELVGITAGEAANPRKTVPRAINKVVFRI    350

CAN1               329 LTFYIGSLLFIGLLVPYNDPKLTQSTSYVSTSPFIIAIENSGTKVLPHIF    378
LYP1               351 VLFYIMSLFFIGLLVPYNDSRLSASSAVIASSPFVISIQNAGTYALPDIF    400

CAN1               379 NAVILTTIISAANSNIYVGSRILFGLSKNKLAPKFLSRTTKGGVPYIAVF    428
LYP1               401 NAVVLITVVSAANSNVYVGSRVLYSLARTGNAPKQFGYVTRQGVPYLGVV    450

CAN1               429 VTAAFGALAYMETSTGGDKVFEWLLNITGVAGFFAWLFISISHIRFMQAL    478
LYP1               451 CTAALGLLAFLVVNNNANTAFNWLINISTLAGLCAWLFISLAHIRFMQAL    500

CAN1               479 KYRGISRDELPFKAKLMPGLAYYAATFMTIIIIIQGFTAFAPKFNGVSFA    528
LYP1               501 KHRGISRDDLPFKAKLMPYGAYYAAFFVTVIIFIQGFQAFCP-FKVSEFF    549

CAN1               529 AAYISIFLFLAVWILFQCIFRCRFIWKIGDVDIDSDRRDIEAIVWEDHEP    578
LYP1               550 TSYISLILLAVVFIGCQIYYKCRFIWKLEDIDIDSDRREIEAIIWEDDEP    599

CAN1               579 KTFWDKFWNVVA                                          590
LYP1               600 KNLWEKFWAAVA                                          611
```

Figure S2: Multiple sequence alignment of Can1 and Lyp1. Sequence analysis is made by the application 'Needle' of EMBL-EBI[68]**.** Boxed are the regions annotated as extracellular loops. For Can1, this is based on the 3D-model made by Ghaddar et al on the basis of AdiCl[12]. For Lyp1, this is based on the EvFold model presented in this article. Amino acid residues that are annotated in extracellular loops for both Can1 and Lyp1 are colored red and amino acid residues that are annotated in extracellular loops of either Can1 or Lyp1 are colored blue.

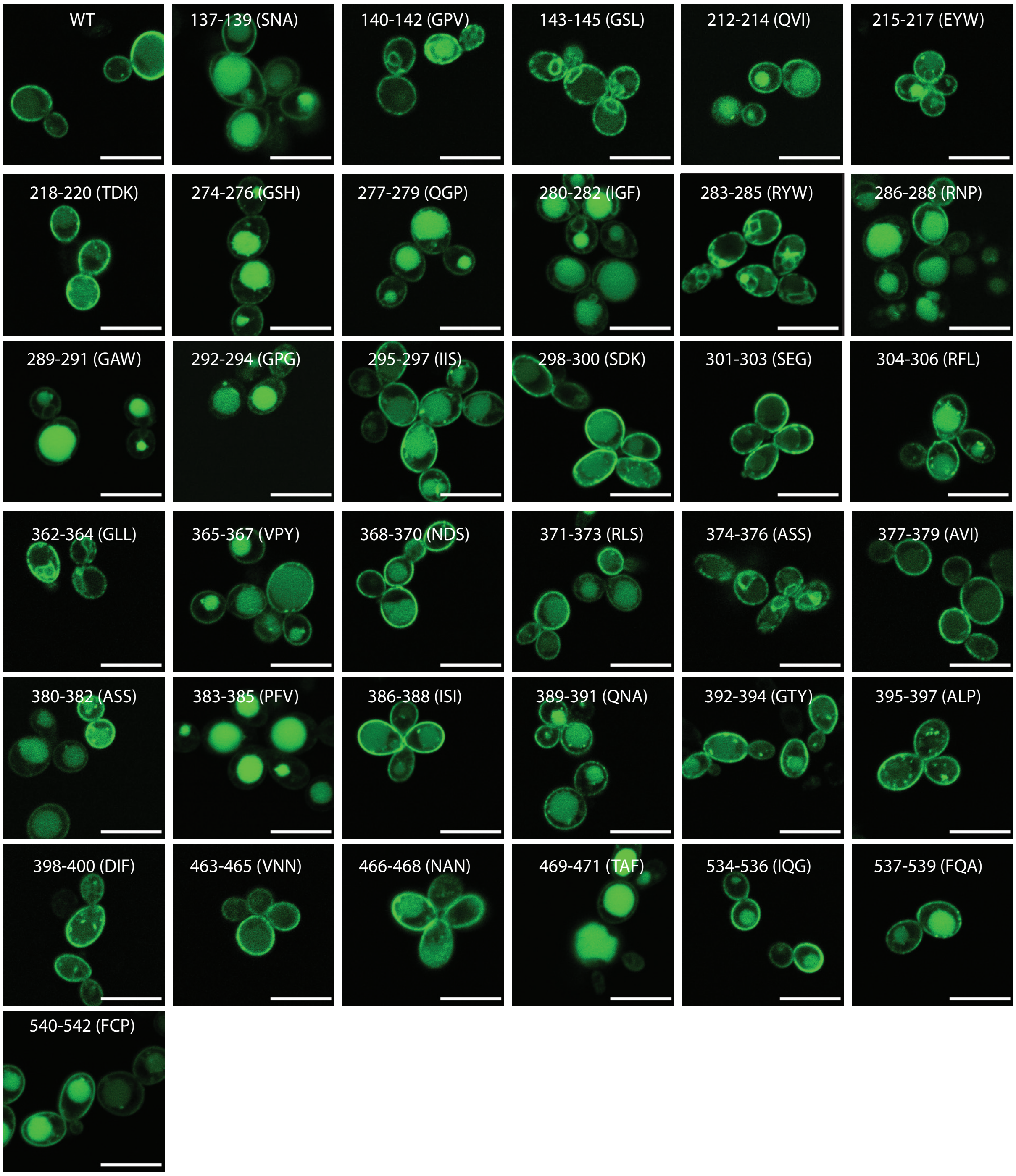

Figure S3: Fluorescence microscopy images of Lyp1 wildtype and mutants. Visualized are Lyp1-GFP molecules in the mid-plane section of the cell. Images are adjusted using the brightness and contrast tool in ImageJ. Scale bars are 10 μM.
